# Formation of β-cyclodextrin complexes in an anhydrous environment

**DOI:** 10.1007/s00894-016-3061-6

**Published:** 2016-08-12

**Authors:** Hocine Sifaoui, Ali Modarressi, Pierre Magri, Anna Stachowicz-Kuśnierz, Jacek Korchowiec, Marek Rogalski

**Affiliations:** 1 Laboratoire de Physico-Chimie des Matériaux et Catalyse, Département de Chimie, Faculté des Sciences Exactes, Université Abderrahmane Mira, Béjaïa, Algeria; 2 Laboratoire de Chimie et de Physique Approches Multi-échelles des Milieux Complexes, Université de Lorraine, 1 Boulevard Arago, Technopole, 57070 Metz, France; 3 K. Guminski Department of Theoretical Chemistry, Faculty of Chemistry, Jagiellonian University, R. Inardena 3, 30-060 Kraków, Poland

**Keywords:** β-Cyclodextrin, Differential scanning calorimetry, Inclusion complexes, Polyaromatics, Organic acids, Molecular modeling

## Abstract

The formation of inclusion complexes of β-cyclodextrin was studied at the melting temperature of guest compounds by differential scanning calorimetry. The complexes of long-chain *n*-alkanes, polyaromatics, and organic acids were investigated by calorimetry and IR spectroscopy. The complexation ratio of β-cyclodextrin was compared with results obtained in an aqueous environment. The stability and structure of inclusion complexes with various stoichiometries were estimated by quantum chemistry and molecular dynamics calculations. Comparison of experimental and theoretical results confirmed the possible formation of multiple inclusion complexes with guest molecules capable of forming hydrogen bonds. This finding gives new insight into the mechanism of formation of host–guest complexes and shows that hydrophobic interactions play a secondary role in this case.

**Electronic supplementary material:**

The online version of this article (doi:10.1007/s00894-016-3061-6) contains supplementary material, which is available to authorized users.

**Graphical abstract:**
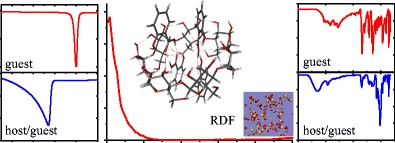
The formation of complexes of β-cyclodextrin with selected *n*-alkanes, polyaromatics, and organic acids in an anhydrous environment is studied by differential scanning calorimetry, IR spectroscopy, and molecular modeling. The results obtained confirm the possible formation of multiple inclusion complexes with guest molecules capable of forming hydrogen bonds and give a new perspective on the mechanism of formation of host-guest complexes.

## Introduction

Many lipophilic drug molecules display low bioavailability that hinder their efficacy [1]. Cyclodextrins, which form stable complexes with numerous molecules, are used to improve aqueous solubility and bioavailability. β-Cyclodextrin (β-CD) is composed of seven glucopyranose units forming a cyclic, cone-shaped cavity with a hydrophilic outer surface and a relatively hydrophobic inner surface [2]. This molecular structure of β-CD allows the formation of complexes with a large variety of organic molecules of different shape and polarity [3–8]. It is widely accepted that β-CD mostly forms inclusion complexes. In aqueous solutions the less polar part of the guest molecule is accommodated by the cavity and the more polar part is exposed to the aqueous environment. The stoichiometry of the complexes, as reported in the literature, is usually 1:1, but 1:2 complexes (i.e., two β-CD molecules per guest molecule) have also been observed [3]. Nevertheless, the interpretation of the complex formation mechanism is not straightforward because of the complexity of the molecular interactions involved. The inclusion process is controlled not only by van der Waals, electronic, hydrogen-bonding and hydrophobic interactions but also by the entropic balance of the dehydration of the cavity and of the guest molecule [9–14]. Therefore, the formation of noninclusion complexes by β-CD with certain guest molecules is also possible. Cyclodextrins may form crystalline and soluble adducts [15–22] as well as low molecular weight organogels [16, 22–26].

Recent experimental results [27, 28] have shown that in water β-CD forms nanometer-sized aggregates of different shape. This lowers the concentration of free cyclodextrin and slows the kinetics of inclusion reactions in aqueous solutions [29, 30]. Moreover, strong hydration of cyclodextrins in an aqueous environment hinders the inclusion reaction. On the other hand it is widely admitted that water plays an important role in the formation of complexes, solubilizing guest molecules and favoring the inclusion of a guest molecule in the cavity through hydrophobic interactions. Thus, both energetic and entropic balance of the replacement of water by guest molecules is decisive for the thermodynamics of inclusion.

In the present study we investigated β-CD complex formation in an anhydrous environment. In these conditions the available volume of the β-CD cavity attains a maximum value, and formation of multiple inclusion complexes of the form *N*:1, with *N* > 1 being the number of guest molecules in the complex, becomes more probable. Moreover, guest molecules are easily stabilized in the cavity by hydrogen bonds formed with hydroxyl moieties of β-CD. The inclusion reaction of selected *n*-alkanes, polyaromatic hydrocarbons, and organic acids was conducted above the melting temperature of the guest compounds with dehydrated β-CD. Progress of the complexation reaction was followed by differential scanning calorimetry [31, 32], and the corresponding value of the complexation ratio, *α*, was established.

The results obtained indicated possible formation of multiple inclusion complexes. Analysis of IR spectra of the complexes supports this hypothesis. The stability and structure of these complexes were determined by quantum mechanics calculations. Molecular dynamics calculations allowed us to establish the geometric conditions of the multiple inclusion complex formation.

## Materials and methods

### Materials

β-CD (CAS no. 7585-39-9), was obtained from Aldrich and was dehydrated at 120 °C under reduced pressure. Naphthalene (CAS no. 91-20-3), dibenzofuran (CAS no. 132-64-9), salicylic acid (CAS no. 69-72-7), benzoic acid (CAS no. 65-85-0), acetylsalicylic acid (CAS no. 69-72-7), decosanoic acid (behenic acid) (CAS no. 112-85-6), *n*-docosane (CAS no. 69-72-7), *n*-octacosane (CAS no.69-72-7), and *n*-hexatriacontane (CAS no. 69-72-7) were from Aldrich. Anthracene (CAS no. 120-12-7) was from Fluka The purity of all chemicals was greater than 99 % w/w, and the purity of benzoic acid was greater than 99.5 % w/w.

### Experimental methods

#### Differential scanning calorimetry experiments

Calorimetric measurements were performed with a TA Instruments differential scanning calorimeter (model 2920 CE). The instrument was calibrated for temperature and heat flow with use of a high-purity indium sample. The uncertainties of temperature and heat measurements were ±0.3 °C and ±0.024 kcal/mol respectively. All of the thermal curves were obtained at a heating rate of 5 °C/min in an inert atmosphere of argon gas (25 mL/min). The baseline was checked before each experiment with use of empty aluminum pans. Differential scanning calorimetry measurements were performed for small samples (2–10 mg) placed in sealed, hermetic aluminum pans. Samples were prepared with a microbalance with an accuracy of about ±0.005 mg. Prepared mixtures contained 50 % w/w of the guest compound and β-CD. Therefore, all samples contained a molar excess of the guest compound. Prepared mixtures were heated in the calorimeter up to temperatures higher than the melting temperature of the guest compound and were maintained at constant temperature for 30 min. After cooling, the sample was equilibrated for 24 h at ambient temperature and the second calorimetric run was performed. The heat necessary to melt the guest compound was determined in both cases. Three measurements were performed for each guest compound. The thermal curves obtained during the third and further runs were very close to the thermal curve obtained during the second run. Therefore, the maximum complexation ratio was reached during the first calorimetric run. During the second run, only the free guest molecule melts. This hypothesis is supported by analysis of the thermal curves of pure guest compounds and those of complexes.

The amount of heat absorbed per mole of guest molecule at the melting temperature is lower in presence of β-CD than the fusion enthalpy because the guest molecules involved in the complex are excluded from the melting process.

Assuming that the guest molecules involved in the complex lost a degree of freedom characteristic for the liquid state, the complexation ratio is proportional to the difference between the molar heat observed in the second calorimetric run and the melting enthalpy of the guest molecule. The calorimetric results (i.e., the energy necessary to melt a crystal of the free guest molecule in the sample) allowed estimation of the inclusion ratio (i.e., the number of guest molecules involved in complex formation per β-CD molecule).

#### IR spectroscopy

IR spectroscopy was used to determine bond changes occurring during complex formation and verify the hypothesis of formation of multiple inclusion complexes. We prepared samples of complexes by grinding the guest compound with β-CD in 4:1 molar ratio either at 25 °C or at the melting temperature of the guest molecule. Fourier transform IR spectra were recorded at room temperature with a PerkinElmer Spectrum One Fourier transform spectrometer, in attenuated total reflectance operating mode, in combination with a KBr beam splitter and a deuterated triglycine sulfate detector with a KBr window.

### Calculations

#### Electronic structure of inclusion complexes

Inclusion complexes of aromatic (rigid) molecules were considered. Dimers (benzene, naphthalene, anthracene, biphenyl, salicylic acid, and benzoic acid) and tetramers (benzene, salicylic acid, and benzoic acid) were also considered. Complexes of β-CD with *n*-alkanes and behenic acid were not investigated because of the high number of rotational degrees of freedom. Calculations were performed with the Gaussian 09 [33] suite of programs. All systems investigated were optimized with the M06-2X functional [34]. The 6-31G(d) basis set was used in the calculations. In contrast to our previous investigations [35] we changed the functional from B3LYP to M06-2X. The hybrid B3LYP potential describes relatively poorly the stacking interactions, and such interactions are important in non-covalently bonded dimers and tetramers. As demonstrated by Zhao and Truhlar [34], the hybrid meta exchange–correlation functional from the M06 suite improved the description of *π*-system thermochemistry, noncovalent interaction energies (*π*–*π* stacking, hydrogen bonding, weak interaction complexes, dispersion-like complexes), and transition metal energetics. The starting geometries (several for each system) were taken from Born–Oppenheimer molecular dynamics simulations. The procedure was the same as in our previous article [36]. In addition, all inclusion complexes were analyzed by the use of quantum theory of atoms in molecules (QTAIM) [37].

Six structures of β-CD were previously located in the gas phase [35]. Four of them had a molecular cavity closed at the lower rim side. Namely, primary hydroxyl groups narrowed the rim by forming a net of hydrogen bonds (alcohol-to-alcohol hydrogen-bond orientation). Depending on the hydrogen donor and acceptor, the left-handed and right-handed orientations were distinguished. The same kind of orientation was observed in the upper rim. These results were consistent with those of Snor et al. [38] obtained with a slightly larger basis set. The existence of hydrogen-bond belts from both sides of the cone is responsible for the rigidity of the β-CD molecule and probably also for low water solubility of all cyclodextrins [39]. Other possible structures of β-CD resulted from internal rotation of the primary hydroxyl groups within the glucopyranose residues. In these structures, alcohol-to-ether hydrogen bonds were formed. Reorientation of hydrogen bonds opened the lower rim. The hydrogen bonds in the upper rim remained unchanged. Such structures were also reported for α-cyclodextrin [40]. It is usually assumed that an open conformation is favored in solution. All previously located structures of β-CD were reoptimized with the M06-2X functional. All of the above-mentioned observations concerning mutual stabilization remained unchanged.

#### Molecular dynamics calculations

The simulation setup included 100 molecules of β-CD and 900 guest molecules. At the beginning of the molecular dynamics calculations all β-CD cavities were empty. The initial geometries of all molecules were taken from quantum-chemical calculations (B3LYP/6-31G*). The molecules were distributed on a rectangular three-dimensional lattice. Each molecule was randomly rotated in space. Calculations were performed with the NAMD2 package [41] and CHARMM force fields [42–44]. The CHARMM general force field and the CHARMM all-atom force field for carbohydrates were used to model guest molecules and β-CD respectively. The applicability of this combination of force fields for modeling inclusion complexes of β-CD was shown in our previous study [36], by comparison with Born–Oppenheimer ab initio molecular dynamics. The total number of particles (*N*), the temperature (*T*), and the pressure (*p*) were kept constant during the simulations. Three-dimensional periodic boundary conditions were used. The particle-mesh Ewald method [45] was used to calculate long-range electrostatic energy with a cutoff value of 12 Å . The same cutoff was applied for van der Waals interactions. The initial configuration of all systems was obtained after 2 ns of *NVT* simulation. The *NpT* simulations with a 1-fs time step were performed for another 40 ns to let the system reach equilibrium. The analysis was performed for a production run of 5 ns (250 simulation frames). Pressure and temperature were set to 1 atm and 137 °C respectively.

## Results and discussion

Measurements and calculations were performed with three families of compounds: long chain *n*-alkanes, aromatic and polyaromatic hydrocarbons, and carboxylic acids. In the case of simple aromatics (benzene, biphenyl), calorimetric measurements were not possible because of the high volatility of these compounds. Nevertheless, simple aromatics are included in the computational part of the study.

### Calorimetric results

**Fig. 1:**
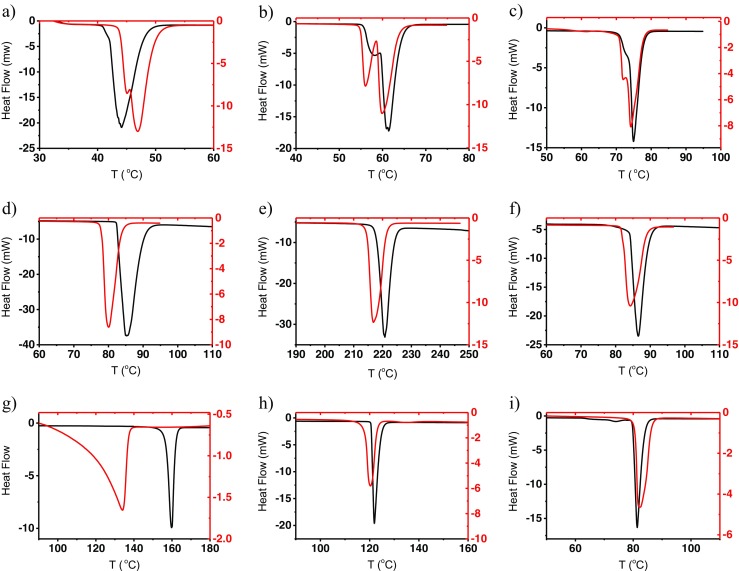
Differential scanning calorimetry thermal curves of pure guest molecules and β-cyclodextrin (β-CD) plus guest binary mixtures. *Black curves* correspond to pure guest molecules. *Red curves* illustrate the second run, where only noncomplexed guest molecules melt. **a** *n*-docosane, **b** *n*-octacosane, **c** *n*-hexatriacontane, **d** naphthalene, **e** anthracene, **f** dibenzofuran, **g** salicylic acid, **h** benzoic acid, and **i** behenic acid

Examples of the resulting thermal curves are shown in Fig. 1. For each guest molecule the black thermal curve corresponds to the melting of the pure guest compound and the red thermal curve corresponds to the second run of the sample composed of the guest molecule, β-CD, and the complex formed during the first run.

Comparison of the black and red thermal curves indicates the formation of inclusion complexes and changes of the melting mechanism induced by the presence of β-CD. In the case of three odd *n*-alkanes (Fig. 1a–c), the premelting phenomena are sharply separated from the fusion itself. The starting temperature of chain-end segments’ disordering does not change in the presence of β-CD with *n*-octacosane and *n*-hexatriacontane but is several degrees Celsius lower in the case of *n*-docosane. This observation indicates that even those molecules of *n*-alkanes that do not form complexes are influenced by the presence of β-CD. This is probably due to the ordering effect of the alkyl chains of *n*-alkanes partly immobilized in the β-CD cavity. The complexation ratio, *α*, corresponding to the number of guest–host complexes per β-CD molecule, is given in Table 1. In the case of *n*-alkanes, the formation of 1:1 complexes is widely accepted [46, 47]. Values of *α* ranging from 0.16 to 0.40 indicate that roughly 30% of β-CD molecules were bonded to *n*-alkanes.

**Table 1:** Number of moles of the guest molecule bonded to 1 mol of β-cyclodextrin (*β-CD*) at the melting temperature of the guest molecule as obtained from differential scanning calorimetry experiments

Guest molecule	Number of moles of the guest molecule complexed by 1 mol of β-CD	
	Aqueous solution	At the melting temperature of the guest molecule
*n*-Alkanes		
*n*-Docosane		0.40
*n*-Octacosane		0.16
*n*-Hexatriacontane		0.19
Aromatics		
Biphenyl	0.84^a^	
Benzene	1.90^a^	
Polyaromatics		
Naphthalene	0.28,^b^ 1.20^a^	0.66
Anthracene	0.46^b^	1.19
Dibenzofuran		0.11
Acids		
Salicylic acid		3.99
Benzoic acid	0.26^b^	3.55
Behenic acid		0.22

^a^Comparison with experimental data obtained with the precipitation method

^b^Comparison with results calculated with the complexation constant in aqueous solutions according to Rekharsky and Inoue [3]

In the case of polyaromatics (Fig. 1d–f) the melting temperature of the complex is 2–3 °C lower than the melting temperature of pure hydrocarbons. This could be explained by a small amount of β-CD dissolved in the aromatic liquid phase at this temperature. The values of *α* obtained for polyaromatics are given in the third column in Table 1. For comparison, in the second column literature data are presented. In contrast to the present study, these data correspond to aqueous solution, so the comparison may be only approximate. The complexation ratios observed with naphthalene and anthracene were 0.66 and 1.19 respectively, much higher than with *n*-alkanes. In the case of anthracene, all β-CD molecules contained a guest molecule. A value of *α* greater than 1 indicates that 2:1 complexes are formed; *α* = 1.19 corresponds to 60% yield of the inclusion reaction. In the case of naphthalene, it is impossible to draw conclusions about the stoichiometry of the complexes. Sanasema et al. [48] give *α* > 1, which seems consistent with the results obtained for anthracene. Assuming formation of a 2:1 complex, the reaction yield would be only 33%.

It can be seen that the inclusion ratio in an anhydrous environment is nearly twice the inclusion ratio in aqueous solution as calculated with the equilibrium constant reported by Rekharsky and Inoue [3]. On the other hand, Sanasema et al. [48] determined complexation ratios of a series of hydrocarbons from precipitation experiments, and reported *α* equal to 0.89, 1.3, 0.84, 1.9, and 1.2 for *n*-hexane, cyclohexane, biphenyl, benzene, and naphthalene respectively. These values are significantly higher than those obtained with equilibrium constants. The values of *α* for benzene, cyclohexane, and naphthalene suggest the formation of 2:1 complexes of β-CD with cyclic hydrocarbons. The stabilization of anthracene is probably less efficient in an aqueous environment because of the presence of water molecules solvating hydroxyl groups of β-CD. Still, similarly to our results, a greater value of *α* is observed for anthracene than for naphthalene.

The inclusion ratio observed with dibenzofuran is much lower. The low value of *α* observed in this case may be explained by aggregation of dibenzofuran. The presence of a heteroatom favors formation of stacked or planar dimers and multimers, which are too voluminous to fit into the β-CD cavity. Calculations confirmed the possible existence of these structures.

Mixtures containing benzoic and behenic acids (Fig. 1h, i) display a small change of the melting temperature of about 2–3 °C, but in the case of salicylic acid (Fig. 1g) this difference is much higher and the maximum of the melting peak appears 45 °C before the melting temperature of pure acid. The only acceptable explanation is significant solubility of β-CD in liquid salicylic acid. Although the complexation ratio of behenic acid was close to that observed with *n*-alkanes, the values of *α* obtained with benzoic and salicylic acids where close to 4, suggesting that four guest molecules might be included in one molecule of β-CD.

The formation of a 4:1 complex necessitates either the inclusion of two stacked dimers or stabilization of the 2:1 complex by two complementary acid molecules external to the cavity. Carboxylic acids easily form stable dimers, but the possibility of dimer inclusion was not confirmed. The formation of 4:1 complexes is studied further with IR spectra of the complexes and by means of molecular modeling.

### IR spectroscopy

**Fig. 2:**
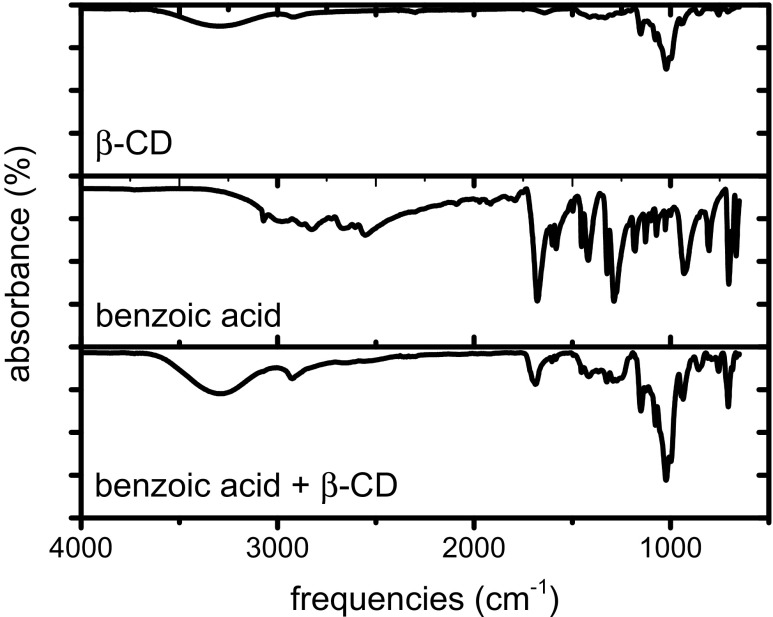
IR spectra obtained with pure β-CD, benzoic acid, and the corresponding 4:1 guest to β-CD mixture

To confirm the hypothesis of 4:1 complex formation, we studied IR spectra of solid complexes of β-CD with benzoic acid and salicylic acid synthesized as described in “IR spectroscopy.” The main difference in the spectra of the acids was observed in the frequency range from 2500 to 3200 cm^−1^, corresponding to aromatic hydrogen vibrations. These vibrations are highly attenuated in mixtures, which is probably due to the inclusion of the acid aromatic rings in the cavity and the formation of multiple hydrogen bonds as described in “Electronic structure of inclusion complexes.” The samples were prepared with a 4:1 guest to β-CD ratio corresponding to the supposed formation of a 4:1 inclusion complex. In the case of a high yield of the complexation reaction, the frequency attenuation of aromatic hydrogen vibrations would be maximal. Total disappearance of the vibrations bands in this frequency range suggests that the total amount of the acid reacted, forming a 4:1 complex. Fig. 2 presents spectra obtained with pure β-CD, benzoic acid, and the corresponding mixture. Nearly total attenuation was observed in this case. For salicylic acid, the spectrum was partly attenuated (shown in Fig. S1). In this case, the yield of the complexation reaction was probably lower. Consequently, the attenuation ratio might be considered as confirmation of multiple inclusion formation and as a tool to estimate the yield of this reaction.

### Molecular modeling

#### Structure of inclusion complexes

**Fig. 3:**
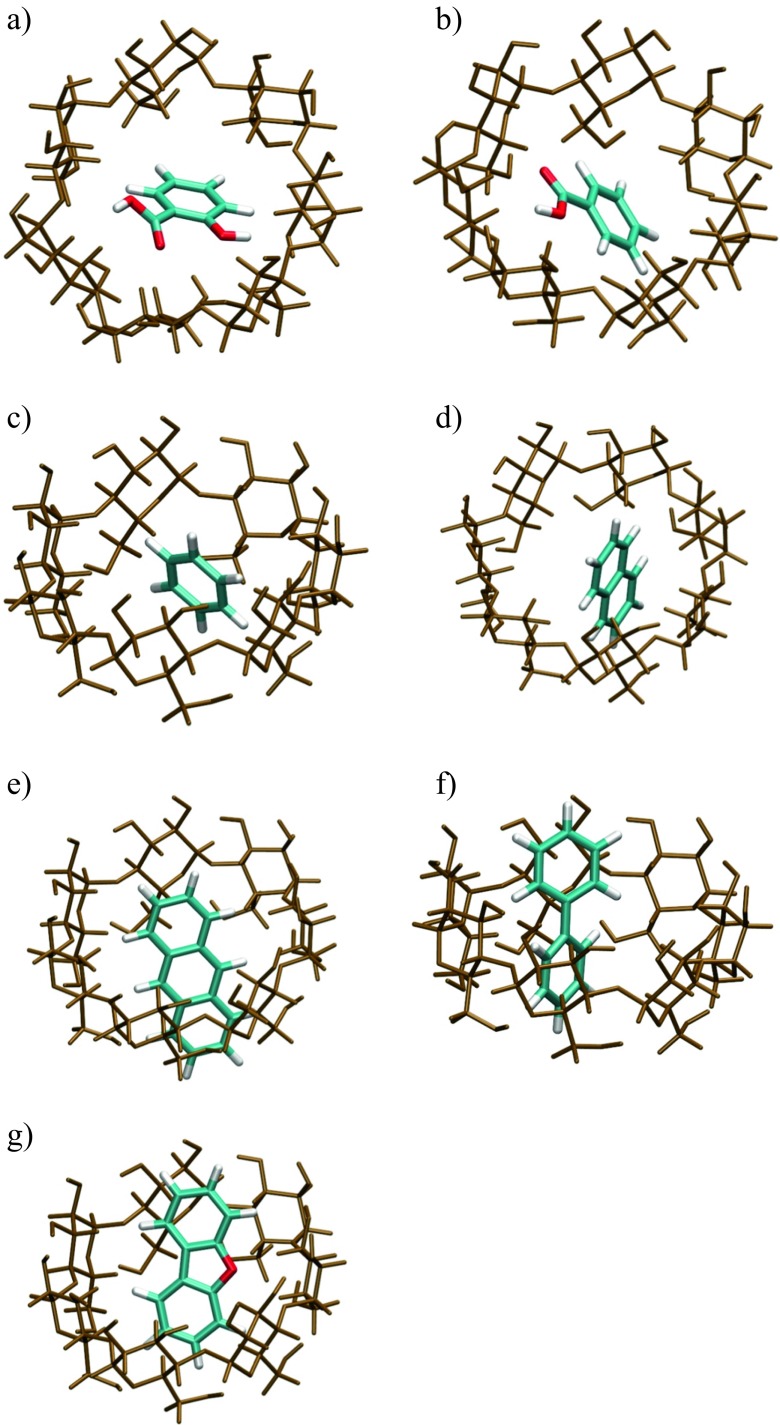
The inclusion complexes of open structures of β-CD with various guest molecules located at the M06-2X/6-31G(d) level of theory: **a** salicylic acid, **b** benzoic acid, **c** benzene, **d** naphthalene, **e** anthracene, **f** biphenyl, and **g** dibenzofuran

**Fig. 4:**
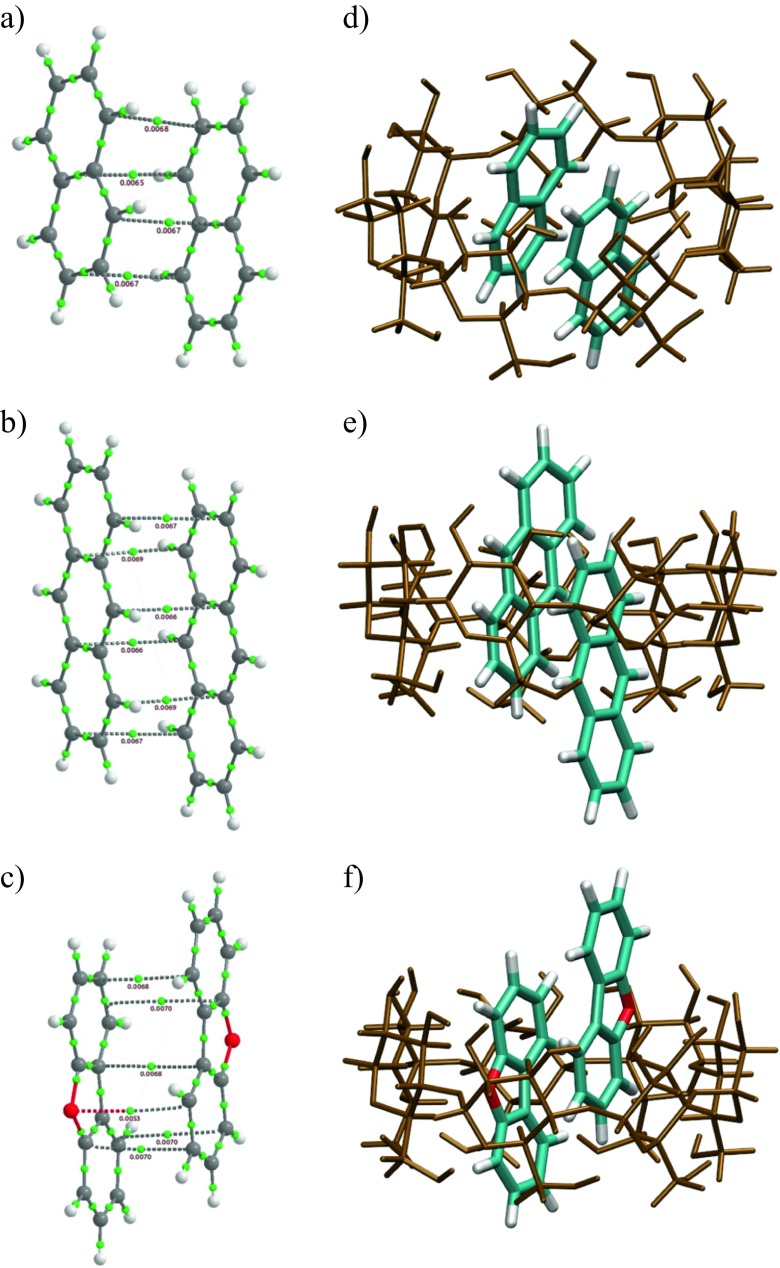
The arrangement of molecules in the isolated stacked dimer of naphthalene (**a**), anthracene (**b**), and dibenzofuran (**c**) together with complex of the open β-CD with a stacked dimer of naphthalene (**d**), anthracene (**e**), and dibenzofuran (**f**)

**Fig. 5:**
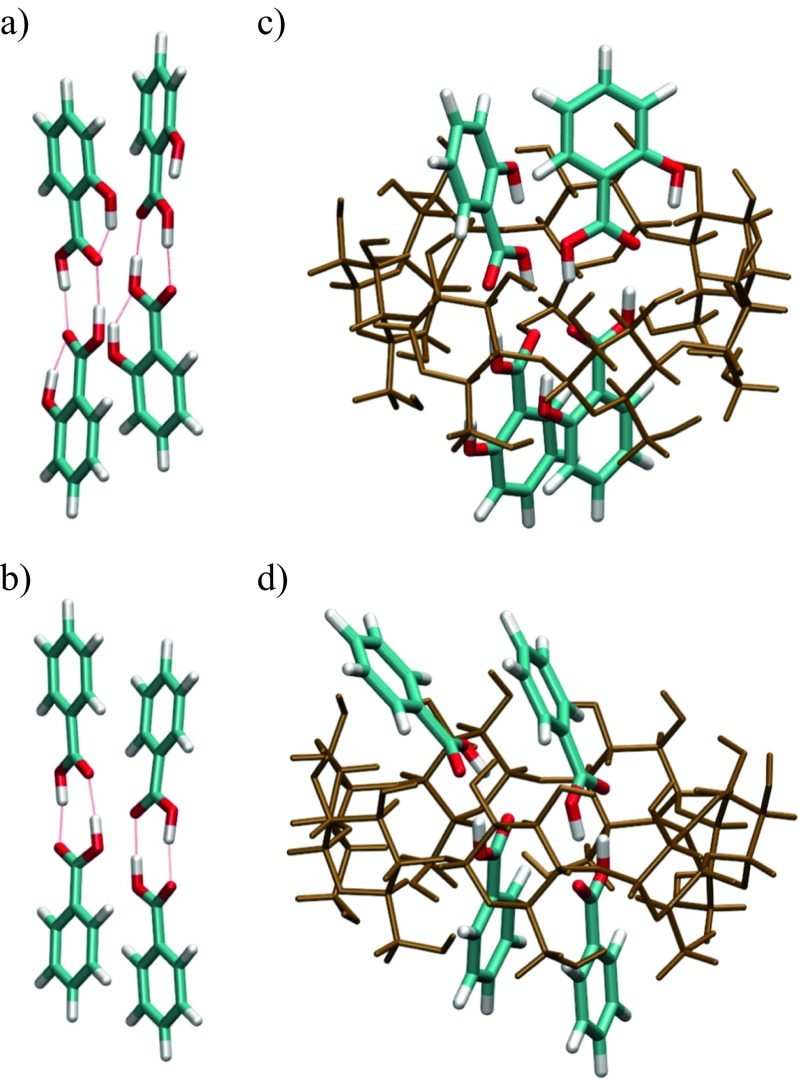
The structure of stacked dimers of salicylic (**a**) and benzoic (**b**) acids. The structures of two acid dimers in the open structure of the β-CD 4:1 complex: **c** salicylic acid and **d** benzoic acid

Interaction energies of inclusion complexes defined as Δ*E*_int_  =  *E*_β -CD/*X*_  −  *E*_β- CD_  −  *E*_*X*_ (where *X* is an aromatic system) are summarized in Table 2. For each entry a few values are reported. These energies correspond to different stoichiometries. In the second column the ratio of β-CD to the guest molecule is 1:1. In the third and fourth columns this ratios are 1:2 and 1:4 respectively. The interaction energies of all the inclusion complexes are negative. It other words, the inclusion complexes are more thermodynamically stable than the separate reactants. The high interaction (stabilization) energies observed with aromatic acids were due to the formation of hydrogen bonds. The geometric structures of all the host–guest complexes are depicted in Figs. 3, 4, and 5. The stoichiometry of the host to guest is 1 (Fig. 3), 2 (Fig. 4), and 4 (Fig. 5) respectively. To understand the main source of stabilization, QTAIM [37] was applied to all inclusion complexes. This method is commonly used to identify intermolecular interactions. It has been successfully applied in characterizing, for example, conventional and unconventional hydrogen bonds [49–51] or both polar [52–55] and nonpolar [56, 57] dihydrogen bonds. A number of topological properties have been shown to correlate with the strength of intermolecular interaction [58–60]. In particular, the existence of a bond critical point (BCP) and a bond path between two atoms is a manifestation of a bonding contact. Further classification can be performed by analysing the value of the electron density at the BCP, *ρ*_BCP_.

**Table 2:** Interaction energy (kcal mol^−1^), Δ*E*  =  *E*_β -CD/*X*_  −  *E*_β -CD_  −  *E*_*X*_ (*X* is a guest molecular system), calculated at the M06-2X/6-31G(d) level of theory

Guest molecule, *X*	β-CD–*X*_*N*_ inclusion complexes		
	*N* = 1	*N* = 2	*N* = 4
Aromatics			
Biphenyl	−18.0	−30.1	
Benzene	−13.0	−21.4	−26.4
Polyaromatics			
Naphthalene	−15.3	–17.5	
Anthracene	−20.0	–19.4	
Dibenzofuran	−19.2	−26.2	
Acids			
Salicylic acid	−30.3	−30.0	−47.9
Benzoic acid	−17.1	−26.4	−36.2

Molecular graphs obtained from QTAIM analysis for selected inclusion complexes are depicted in Fig. S2. The value of *ρ*_BCP_ is given next to a sphere representing each BCP (green ball). Aromatic and polyaromatic molecules are stabilized with bonds formed by oxygen atoms of β-CD and CH moieties of the aromatic ring. The CH moiety in an aromatic ring is more electronegative than CH_2_ in aliphatic chains [61] and one can expect a higher stabilization energy with polyaromatic guest molecules as compared with *n*-alkanes. In the case of aromatic molecules, a system of several hydrogen bonds stabilizes the guest molecule within the cavity. The values of *ρ*_BCP_ at these points range from 0.003 to 0.016 a.u. Therefore, they are contained in the range specified by Koch and Popelier [51] as characteristic for weak hydrogen bonds of the CH⋯O type. Apart from CH⋯O hydrogen bonds, C⋯O, CH⋯HC, and C⋯HC bonding paths are observed. Here, the first and second positions in each pair correspond to guest and host molecules respectively. The axial hydrogen atoms of β-CD directed toward the cavity (H3 and H5) are involved in CH⋯HC and C⋯HC contacts. The CH⋯C contacts cover a distance range similar to that of the CH⋯O contacts. The CH⋯HC and C⋯O bond paths are shorter and longer than CH⋯O bond paths respectively. In all three cases, *ρ*_BCP_ is lower than for CH⋯O interactions; therefore, the latter interactions can be regarded as the most important source of stabilization.

Polyaromatic hydrocarbons, naphthalene, anthracene, and dibenzofuran, can form *π*-stacked systems. Non-covalently bonded dimers are mainly stabilized by weak dispersion energy. This energy, by definition, is not taken into account at the Hartree–Fock level of theory. At the density functional theory level, most functionals, including the most popular B3LYP, describe stacking interactions fairly well. The post-Hartree–Fock methods are appropriate to handle this problem, but unfortunately they are very expensive because of an unfavorable scaling behavior. Recent second-order Møller–Plesset perturbation theory calculations by Yurtsever [62] indicate that the stablest conformation for parallel stacked dimers is not exactly aligned, with a shift of about 1 Å. The distance between stacked anthracene molecules was 3.7 Å, and it was demonstrated that interaction energies were strongly dependent on the basis set used. The interaction energy for a stacked naphthalene dimer, calculated by Tsuzuki et al. [63] using large-scale calculations, was 3.78 kcal mol^−1^ for a monomer separation of 3.8 Å. These results indicate that the cavity of the β-CD molecule is too small for inclusion of dimers of naphthalene, anthracene, or dibenzofuran.

At the M06-2X/6-31(d) level of theory, the stacked conformers are stable. The located structures (QTAIM molecular graphs) are shown in Fig. 4a–c. One can observe four C⋯C bonding paths between naphthalene molecules. The number of bonding paths increases to six for anthracene (C⋯C paths) and dibenzofuran (C⋯C and C⋯O paths) molecules. Anthracene molecules in the dimer are shifted along the *z* axis by 3.3 Å and they are not aligned (shifted along *x* and *y* axes by 1.15 and 1.13 Å). In stacked naphthalene the same separation between naphthalene molecules (3.3 Å) as in the anthracene dimer was obtained. The long axes of the molecules are rotated by 9.6° and shifted from perfect alignment by 1.4 Å (*x* axis) and 1.5 Å (*y* axis). The interaction energies are higher than those reported in literature and are 7.0 and 10.8 kcal mol^−1^ for naphthalene and anthracene respectively. This difference can be attributed to the higher-level methods and larger basis sets used in the studies reported in the literature [62, 63]. In addition, the energies from our study can be further reduced if the basis set superposition error is taken into account. The inclusion complexes investigated in the current study are too big for high-level ab initio calculations. In the case of dibenzofuran, the stabilization of the stacked dimer is 2.9 kcal mol^–1^. This interaction energy encouraged us to check the existence of 2:1 complexes (naphthalene–β-CD, anthracene–β-CD, and dibenzofuran–β-CD). The stacked dimers were inserted into the cavity. The inclusion complexes obtained are shown in Fig. 4d–f. The calculated stabilization energies of the stacked dimers are −17.5, −19.4, and −26.2 kcal mol^−1^ (Table 2). The energy gain due to inclusion of the second guest molecule is smaller compared with the first one. This is due to unfavorable dimer compression forced by the cavity size. The separation between anthracene molecules drops to 3.0 Å. In addition, the long axes of the molecules are twisted by 28.6° and shifted by 2.9 Å along the *x* axis and 1.6 Å along the *y* axis. In other words, one of the guest anthracene molecules is slightly shifted out of the cavity. The corresponding reduced mean square deviation (RMSD) is 1.35 Å. The naphthalene dimer inside the cavity is less distorted than the anthracene dimer. The long axes of naphthalene molecules are rotated by 10.4° and the molecules are shifted by 1.4 and 1.5 Å in the *x* and *y* directions respectively. The RMSD is 1.05 Å. Again, the molecules are more compressed as compared with the isolated naphthalene dimer. These observations explain the lack of additional stabilization after insertion of the second guest molecule into the cavity. It is thought that polyaromatics can form complexes with one or more molecules external to the cavity. The capability of several polyaromatic hydrocarbons to form a stack outside the cyclodextrin cavity was reported by Rizkov et al. [16]. The capability to form external/internal dimers depends on the energetic and geometric properties of stacked molecules. Stacked molecules of dibenzofuran are separated by 3.3 Å. The long axes are rotated by 15°. In addition, the molecules are displaced from perfect stacking. The RMSD is 0.67 Å. However, dibenzofuran can form a planar dimer stabilized by two hydrogen bonds (CH⋯O hydrogen bonds). The interaction energy of such a planar dimer is 9.7 kcal mol^−1^. This may have an impact on the complexation ratio. The finding that polyaromatic hydrocarbons form 2:1 dimers with one molecule external to the cavity explains the low values of *α* obtained from calorimetric measurements. The guest molecule external to the cavity has more degrees of freedom and the calorimetric response is different as compared with the molecules stabilized in the cavity.

Additionally, we performed calculations for systems with single aromatic rings—namely, benzene and biphenyl molecules. The highest stabilization was observed for biphenyl (Table 2). The energy gain due to the insertion of the second guest molecule is more pronounced as compared with the polyaromatic systems. This can be explained by higher flexibility of the biphenyl molecule. In the case of benzene, stacking interactions are too small to keep such a conformation inside the cavity. The stabilization of the two benzene molecules is almost twice as large as that of one molecule. The final structure is no longer a stacked dimer (intermediate between a T-shaped structure and a planar structure).

To check the existence of 4:1 complexes of β-CD with salicylic and benzoic acids, additional calculations were performed. Namely, two stacked dimers of salicylic acid or benzoic acid were inserted into the cavity of the open form of β-CD. The structures of both stacks and 4:1 complexes are shown in Fig. 5. The configurations of stacked dimers of salicylic acid and benzoic acid are shown in Fig. 5a and b respectively and those of 4:1 complexes of the two acids with β-CD are shown in Fig. 5c and d. The interaction energies obtained were −36.2 and −47.9 kcal mol^−1^ for benzoic acid and salicylic acid respectively. The interaction energies were computed with respect to β-CD and the two stacked dimers shown in Fig. 5a and b.

#### Molecular dynamics results

Molecular dynamics calculations were used to estimate the number of guest molecules, *α*, in the β-CD cavity at 137 °C, which is the temperature roughly corresponding to the experimental conditions of the complexation reactions performed in calorimetric experiments. To estimate *α* we computed the radial distribution of the center of mass of guest molecules around the center of mass of the host molecules. Such a radial distribution function (RDF) is a useful quantity for describing the structure of the complex system. The three-dimensional RDF was computed as follows:

1 $$ g(r)=N(r)/4\uppi {r}^2\rho \delta r, $$

where *N*(*r*) is number of guest molecules in a spherical shell at a distance *r* and thickness *δ r* from a reference center of mass of cyclodextrin, and *ρ* is the number density calculated as the ratio of the number of molecules to the volume of the sphere.

**Fig. 6:**
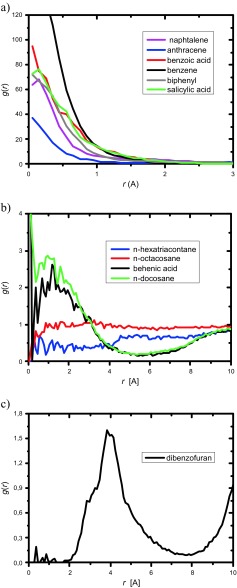
Radial distribution function illustrating the position of the center of mass of guest molecules around the center of mass of the host (cyclodextrin) molecule: **a** aromatic-containing systems, **b** *n*-alkane-containing systems, and **c** dibenzofuran-containing system

The computed RDFs are shown in Fig. 6. Figure 6a presents RDFs for aromatics, polyaromatics, benzoic acid, and salicylic acid. The RDF plots obtained are different from typical RDFs. The highest value of the RDF is observed near the reference center of mass of cyclodextrin. The center of mass of cyclodextrin is located inside the cavity; therefore, these values of the RDFs indicate a high inclusion ratio. A direct comparison among aromatic molecules is not possible since the number density appearing in Eq. 1 is different for different systems. The RDFs of *n*-alkanes and dibenzofuran are shown in In Fig. 6b and c respectively. Their qualitative behavior is different from the behavior shown in Fig. 6a. In the case of *n*-docosane, the inclusion is still important. The RDF inside the cavity (*r* less than approximately 4 Å) has higher values than outside the cavity. For docosane the value of the RDF inside the cavity is almost the same as outside the cavity. For *n*-octacosane the value of the RDF outside the cavity is higher than that inside the cavity. The curves are not as smooth as those in Fig. 6a. Such behavior is connected with huge conformational freedom of *n*-alkanes. The RDF computed for dibenzofuran (Fig. 6c) behaves differently from the RDFs shown in Fig. 6a and b. The maximum of the RDF is shifted toward higher *r* values. Such behavior indicates that dibenzofuran prefers external-type complexes. The size of the molecule is probably responsible for such behavior. The RDF plots qualitatively explain the results given in Table 2.

The inclusion number can be obtained by integration of the RDF. The results obtained are reported in Table 3. Two values are reported: one (*α*_min_) corresponds to the cavity size, and the other (*α*_max_) corresponds to the cavity size extended by 3 Å. We decided to extend the cavity size since one or more molecules in stacked dimers (Fig. 4) or tetramers (Fig. 5) may be external to the host molecule. The results obtained are in qualitative agreement with those in Table 2.

**Table 3:** The number of guest molecules, *α*, in the β-CD cavity at 137 °C obtained from molecular dynamics simulations; *α*_min_ values correspond to the β-CD cavity dimension, whereas *α*_max_ values correspond to extension of the cavity dimension by 3 Å

Guest molecule	α_min_ (*r* = 4 Å)	α_max_ (*r* = 7 Å)
*n*-Alkanes		
*n*-Docosane	0.21	0.33
*n*-Octacosane	0.16	0.32
*n*-Hexatriacontane	0.07	0.35
Aromatics		
Biphenyl	0.70	0.80
Benzene	0.60	0.80
Polyaromatics		
Naphthalene	0.68	0.95
Anthracene	0.62	1.00
Dibenzofuran	0.13	0.42
Acids		
Salicylic acid	0.90	1.91
Benzoic acid	0.81	1.65

## Conclusions

The results of the present study show that β-CD may form complexes with hydrocarbons and organic acids in an anhydrous environment. The observed complexation ratio was higher than values obtained in an aqueous environment and suggested the formation of multiple complexes in the case of organic acids and certain polyaromatic hydrocarbons. Quantum chemistry calculations have showed that it is possible to stabilize more than one molecule in the β-CD cavity. The multiple inclusion is stabilized by hydrogen bonds involving hydrogen atoms of the aromatic ring or the carboxylic moiety. Molecular dynamics calculations confirmed the possible formation of inclusion complexes at high temperatures and showed the influence of geometric factors on the formation of inclusion complexes. Therefore, in water solutions, the multiple inclusion is hindered by water molecules solvating the inner walls of the β-CD cavity. In addition, molecules having a high propensity to form multimers and aggregates cannot form inclusion complexes as was observed with dibenzofuran. The possibility to form multiple complexes explains the high yield of the inclusion reaction obtained with certain polar drug molecules. The results obtained with aromatic acids indicate that aggregates of certain guest molecules may be stabilized within the cavity. In the case of polyaromatics, calculations have shown that the guest molecules accommodated in the cavity may stack with one or more external molecules. In this case, the included molecules may induce long-range ordered structures as described by Rizkov at al. [16]. The possibility of multiple inclusion of internal or internal–external type gives new insight into the mechanism of the formation of host–guest complexes by β-CD. It also explains the long-range ordering of polyaromatics in mixtures containing β-CD.

## Electronic supplementary material

Below is the link to the electronic supplementary material.

Fig. S1 IR spectra obtained with salicylic acid and the corresponding 4:1 guest–β-cyclodextrin (β-CD) mixture. (DOCX 154 kb)

Fig. S2 Molecular graphs of salicylic acid–β-CD (**a**) and naphthalene–β-CD (**b**) inclusion complexes. Bond critical points are represented as green spheres. Intermolecular bond critical pointss are supplemented with the electron densities. (DOCX 655 kb)

Fig. S3 Molecular graphs of benzoic acid (**a**), salicylic acid (**b**), and benzene (**c**) tetramers and biphenyl dimer (**d**) Bond critical points are represented as *green spheres*. Intermolecular bond critical points are supplemented with the electron densities. (DOCX 279 kb)
