# Supplementary figures and images for: Formation of β-cyclodextrin complexes in an anhydrous environment
Source: J Mol Model. 2016 Aug 12;22(9):207. doi: 10.1007/s00894-016-3061-6 (PMC4982878; doi:10.1007/s00894-016-3061-6)

| 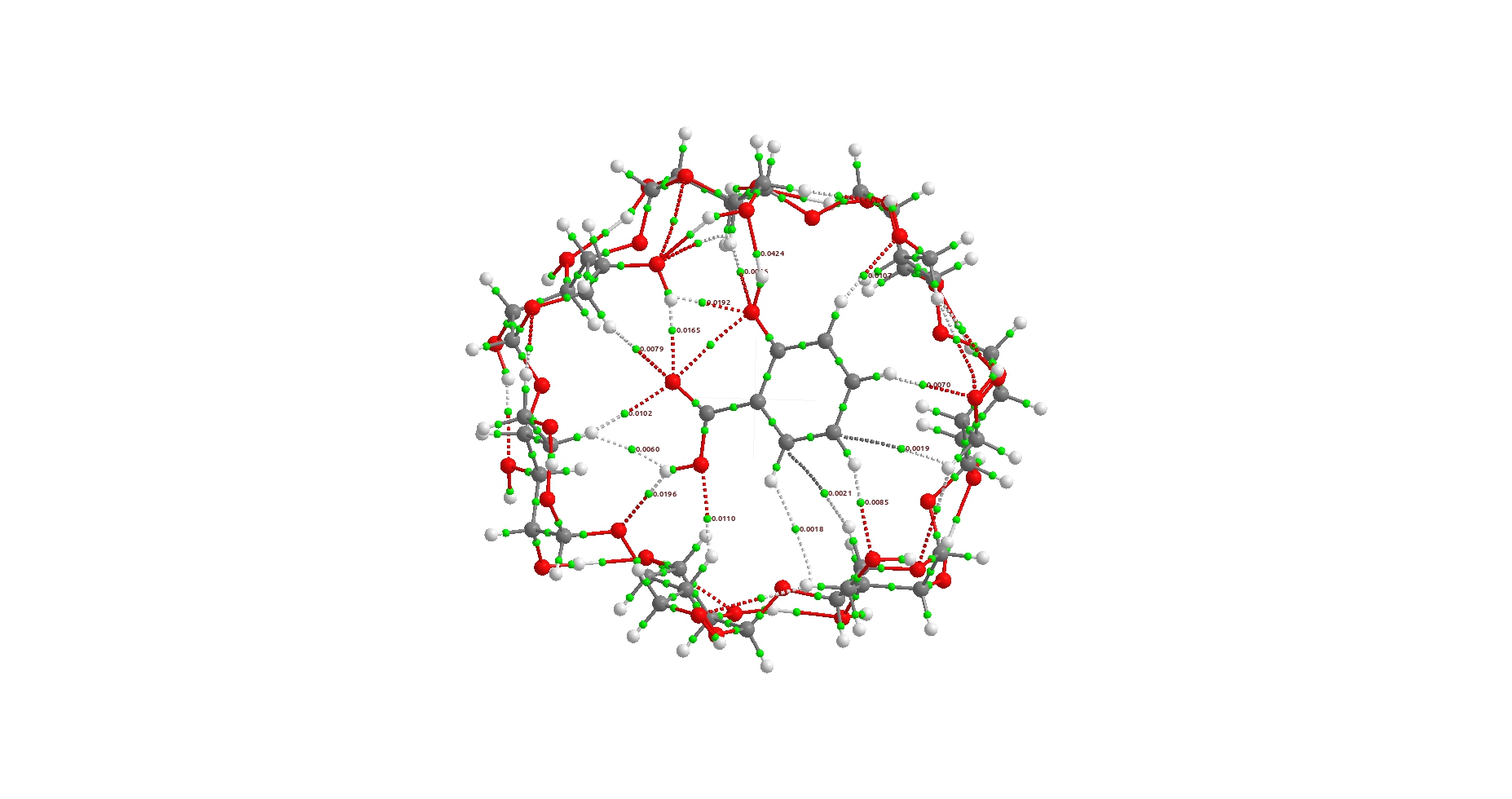  a) |
| --- |
| 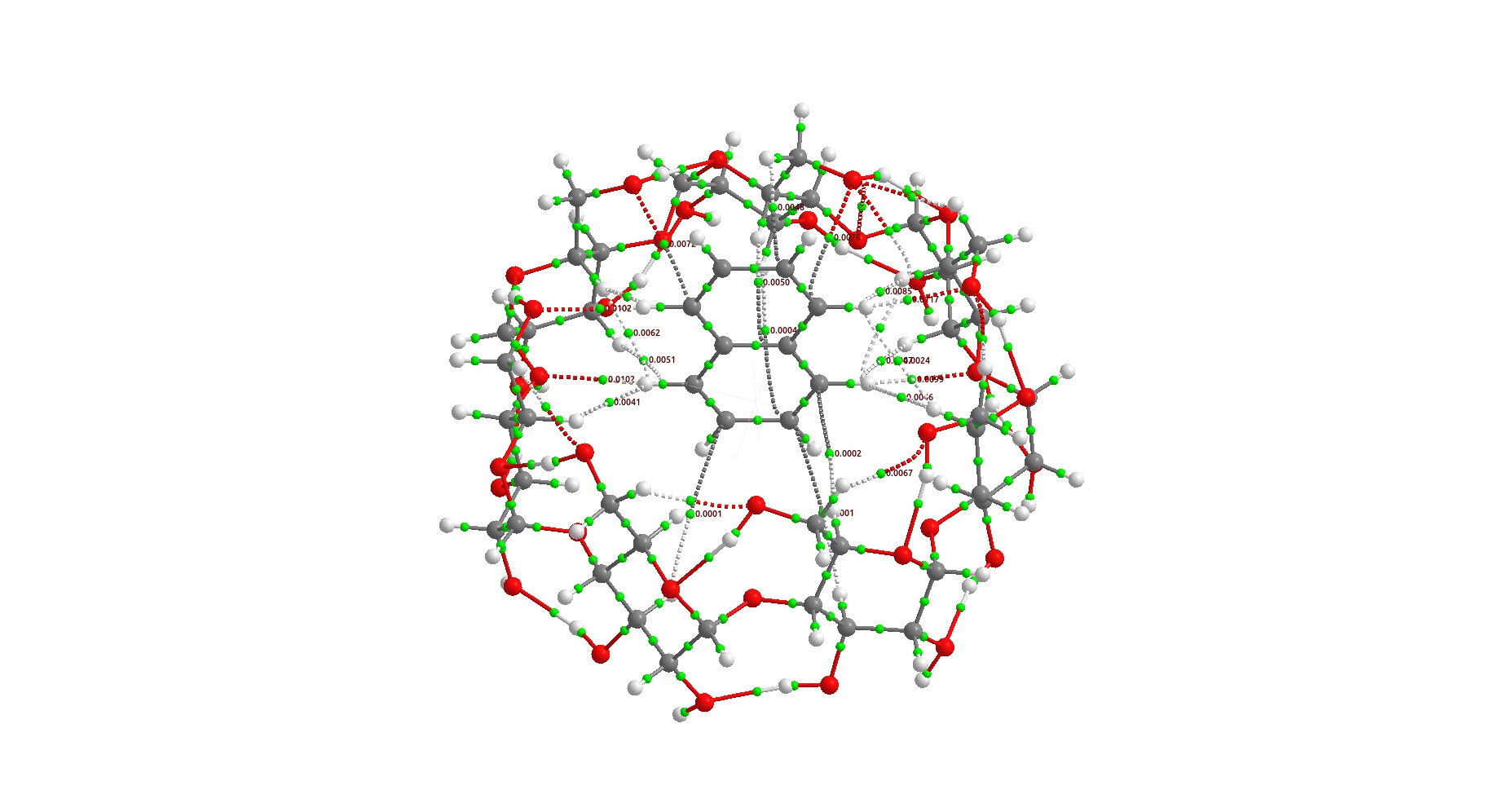  b) |

Figure S2

Supplement: Supplementary file 2 — Molecular graphs of salicylic acid–β-CD (a) and naphthalene–β-CD (b) inclusion complexes. Bond critical points are represented as green spheres. Intermolecular bond critical pointss are supplemented with the electron densities. (DOCX 655 kb) [file 894_2016_3061_MOESM2_ESM.docx]

| 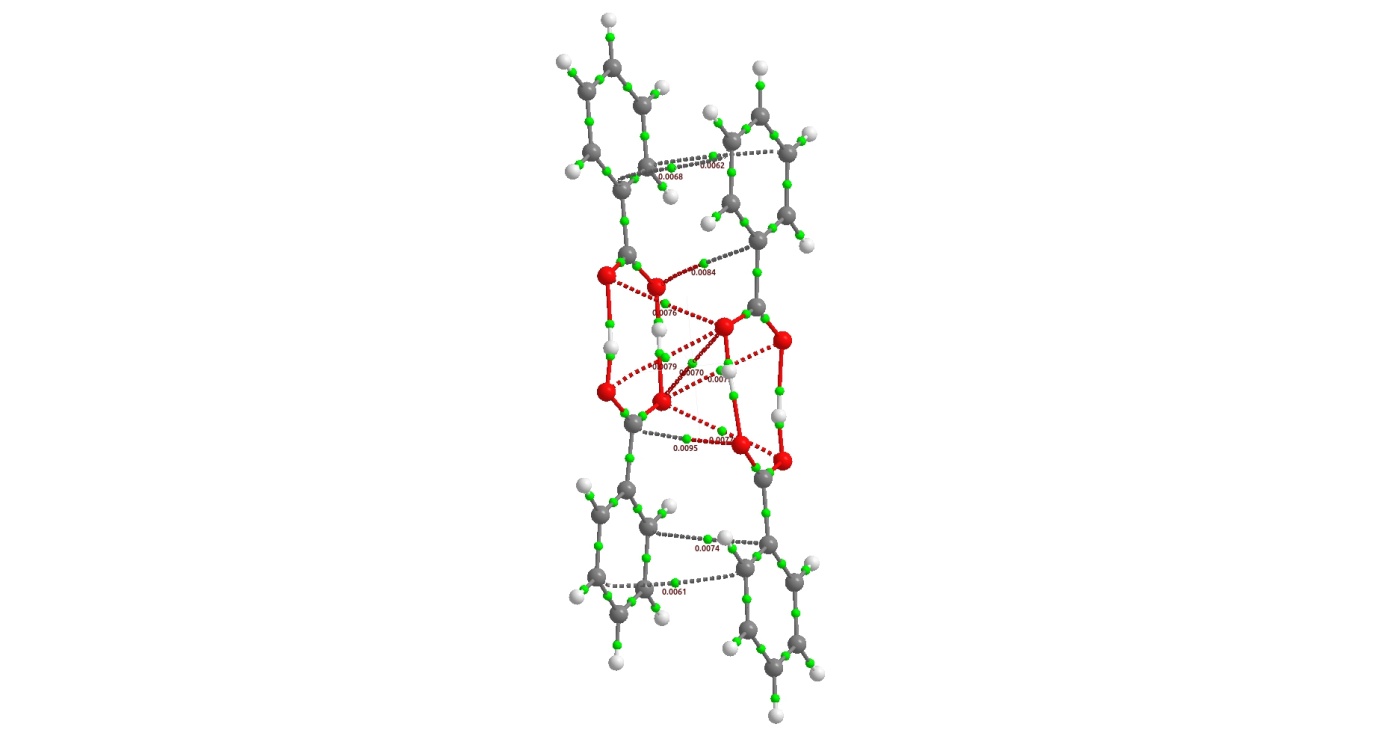  a) | 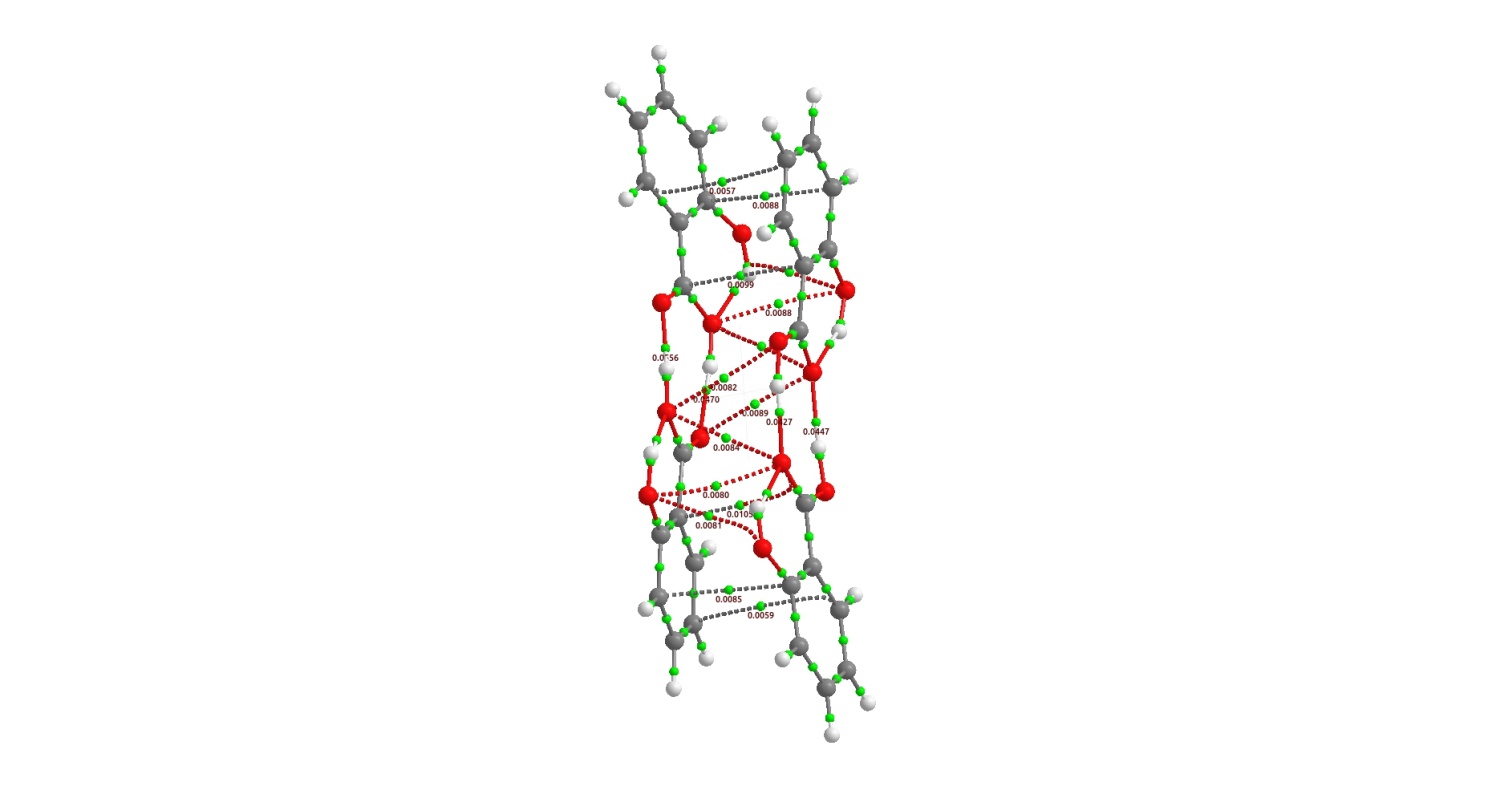  b) |
| --- | --- |
| 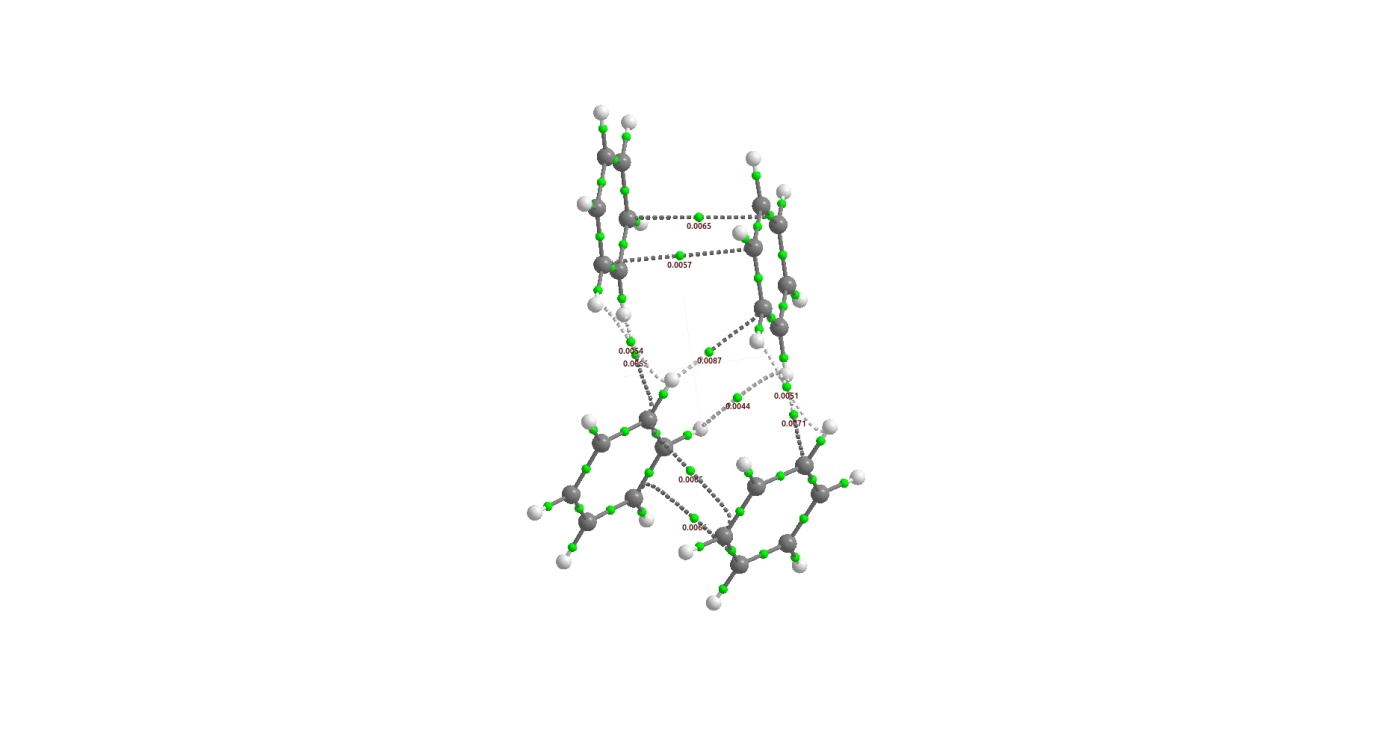  c) | 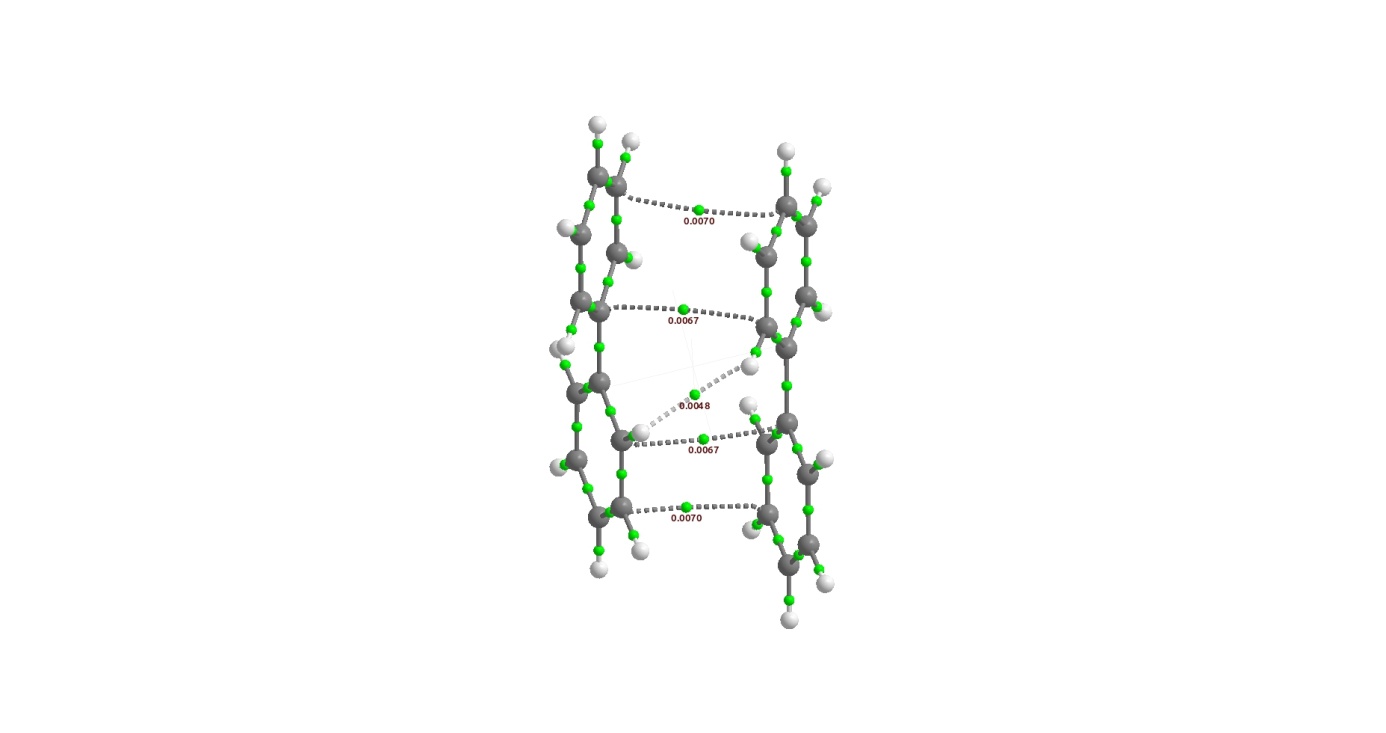  d) |

Figure S3

Supplement: Supplementary file 3 — Molecular graphs of benzoic acid (a), salicylic acid (b), and benzene (c) tetramers and biphenyl dimer (d) Bond critical points are represented as green spheres. Intermolecular bond critical points are supplemented with the electron densities. (DOCX 279 kb) [file 894_2016_3061_MOESM3_ESM.docx]
